# Seasonal Abundance and Diversity of Egg Parasitoids of *Halyomorpha halys* in Kiwifruit Orchards in China

**DOI:** 10.3390/insects12050428

**Published:** 2021-05-10

**Authors:** Gonzalo A. Avila, Juhong Chen, Wenjing Li, Maryam Alavi, Qianqian Mi, Manoharie Sandanayaka, Feng Zhang, Jinping Zhang

**Affiliations:** 1 The New Zealand Institute for Plant and Food Research Limited, Private Bag 92169, Auckland Mail Centre, Auckland 1142, New Zealand; gonzalo.avila@plantandfood.co.nz (G.A.A.); Maryam.Alavi@plantandfood.co.nz (M.A.); Manoharie.Sandanayaka@plantandfood.co.nz (M.S.); 2 MARA-CABI Joint Laboratory for Bio-Safety, Institute of Plant Protection, Chinese Academy of Agricultural Sciences, No. 2 Yuanmingyuan West Road, Beijing 100193, China; juhongchen.lys@gmail.com (J.C.); wenjingli321@gmail.com (W.L.); Q.Mi@cabi.org (Q.M.); F.Zhang@cabi.org (F.Z.); 3 College of Plant Protection, Jilin Agricultural University, No. 2888 Xincheng Street, Changchun 130118, China

**Keywords:** biological control, brown marmorated stink bug, *Trissolcus japonicus*, *Trissolcus cultratus*

## Abstract

**Simple Summary:**

Surveys of egg parasitoids of the brown marmorated stink bug, *Halyomorpha halys,* were conducted in 2018 and 2019 in green-fleshed ‘Hayward’ kiwifruit orchards in Mei County, Shaanxi Province, China, to assess the abundance and diversity of parasitoid communities under two different management systems. Parasitism occurred from June to August and from May to August across the two survey seasons, respectively. Five parasitoid species were recovered from successfully parasitized *H. halys* egg masses, where *Trissolcus japonicus* and *Trissolcus cultratus* were the predominant species. The abundance of *T. japonicus* varied throughout the season in both orchards in both survey seasons. Monthly *T. japonicus* abundance showed a unimodal distribution in 2018, peaking in July. There were two peaks (May–June and August) in the 2019 season. This study provides valuable information on the season-long abundance and diversity of egg parasitoids of *H. halys* in kiwifruit, and most importantly on the abundance and effectiveness, relative to other parasitoids, of *T. japonicus* in kiwifruit orchards.

**Abstract:**

To develop effective and targeted biocontrol tactics for the brown marmorated stink bug, *Halyomorpha halys,* in crop habitats, a good understanding is essential of the abundance and diversity of its parasitoids in different crop habitats in its native range. To obtain information on the egg parasitoid communities of *H. halys* in kiwifruit, surveys using sentinel egg masses were conducted in 2018 and 2019. These assessed the species composition of egg parasitoids of *H. halys* in green-fleshed ‘Hayward’ kiwifruit orchards, and quantified their season-long abundances in orchards under two different management systems. Parasitism was observed from June to August 2018 (mean parasitism: 48%) and from May to August 2019 (mean parasitism: 29%) across the experimental orchards. In total, five different parasitoid species were found across the two surveys seasons in the kiwifruit orchards, *Trissolcus japonicus*, *T. cultratus*, *T. plautiae*, *Anastatus japonicus*, and *Acroclisoides* sp., where *T. japonicus* and *T. cultratus* were the predominant species. Monthly *T. japonicus* abundance data had a unimodal distribution in 2018, peaking in July. There were two peaks (May–June and August) in the 2019 season. Overall, *T. japonicus* was significantly more abundant in the organic orchard than the conventionally managed orchard only in 2018, and its monthly abundance differed significantly in the two orchards in the two survey seasons. Results and their implications for future classical biological control for *H. halys* in kiwifruit are discussed.

## 1. Introduction

The brown marmorated stink bug (BMSB), *Halyomorpha halys* (Stål) (Hemiptera: Pentatomidae), is a highly invasive temperate/subtropical polyphagous pest native to eastern Asia, including China, Japan, and Korea [1]. This pest has successfully established in North America (e.g., the USA, Canada), most of Europe including Russia, and most recently in Chile [2,3,4,5]. BMSB is considered a major agricultural pest, with over 300 host plants recorded worldwide, and is known to cause severe damage to many economically important crops, including vegetables, fruits, and ornamental trees [1,5,6,7]. BMSB is known to cause extensive damage to kiwifruit in its native range in China and Korea [8,9,10], and also in countries in its adventive range, such as Italy [11,12] and Greece [13]. BMSB-associated kiwifruit damage in its adventive range is estimated to be over 30% in kiwifruit production [10,12]. In New Zealand, BMSB is considered a significant economic threat, potentially jeopardising its multibillion-dollar export market for fresh produce (e.g., kiwifruit, apples). Although not yet known to be present in New Zealand, there is a high risk of entry and establishment of BMSB as it has been repeatedly intercepted at the border in recent years [14].

Several enemies of BMSB, including arthropod predators, dipteran parasitoids, and hymenopteran egg parasitoids, have been recorded from both its native and adventive range [15,16,17,18,19]. In its native range, eggs of BMSB are attacked by a complex of species, mainly in the genera *Trissolcus* Ashmead (Scelionidae), *Telenomus* Haliday (Scelionidae), *Ooencyrtus* (Encyrtidae), *Acroclisoides* (Pteromalidae), and *Anastatus* (Eupelmidae) [18,20]. Amongst all reported natural enemies of BMSB, the egg parasitoid *Trissolcus japonicus* (Ashmead) is regarded as the most promising biocontrol agent to be used in a classical biocontrol programme against BMSB in its adventive range [18,21,22,23,24]. The recent discovery of adventive populations of *T. japonicus* in the USA, Canada, Switzerland and Italy [25,26,27,28,29] is expected to mark the starting point for the implementation of classical biological control programmes in these countries. Adventive populations of *T. japonicus* are now being reared and redistributed in Canada, several US states, and in Italy, where the mass release plan was officially started in 2020 [30]. In preparation for the potential arrival/establishment of BMSB in New Zealand, a pre-emptive classical biocontrol programme for BMSB using *T. japonicus* was initiated in December 2015 [31]. Approval for a conditional release of *T. japonicus* was granted by the New Zealand Environmental Protection Authority (EPA) in August 2018, should BMSB be detected in the country [31,32].

The diversity of parasitoid and phytophagous host species, as well as their abundance, varies with plant species and, occasionally, cultivar [33]. Therefore, before starting releases of selected natural enemies as part of a biocontrol programme, it is essential to understand how the selected natural enemies’ abundance and potential efficacy may vary between different crops. A good understanding of parasitism of BMSB egg masses over the season in both crop and woodland habitats, and particularly in its native range, is crucial for the development of effective and targeted biocontrol tactics for this pest in different crop habitats (e.g., fruit crops) [34]. The use of sentinel egg masses has been commonly adopted as a monitoring tool in field surveys for assessing the abundance and diversity of egg parasitoids of BMSB [18,34,35,36,37,38]. This approach has proven effective, especially when naturally laid egg masses are not abundant or are challenging to find [39]. A comprehensive four-year study, conducted by Zhang et al. [18] in northern China, assessed field parasitism of sentinel BMSB egg masses in mixed fruit orchards (i.e., peach, cherry, mulberry and Chinese apple) throughout the season. In this survey, *T. japonicus* was found to be the most abundant egg parasitoid species, accounting for over 70% abundance in all four different fruit crops assessed. However, the high rates of observed parasitism may not necessarily translate to other crops, such as kiwifruit. Parasitoid surveys in the USA have detected *T. japonicus* parasitising BMSB egg masses in woodlands habitats [16,29,40,41] and, most recently, in peach orchards [42]. Observed parasitism, however, was relatively low.

We are unaware of any published studies that have characterised BMSB egg parasitoid communities’ abundance and diversity in kiwifruit orchards in BMSB’s native range. Nor have we found any research on their potential impact on BMSB eggs in such orchards, or on the possible differences between communities of kiwifruit orchards with different management systems (conventional/sprayed, and organic/unsprayed). In this two-season study, we conducted surveys to examine the composition of egg parasitoids of BMSB in ‘Hayward’ kiwifruit orchards, and to quantify their season-long abundances in orchards under different management systems. We report the results of egg parasitoid trapping data collected via sentinel egg mass surveys within kiwifruit orchards conducted in Mei County, Shaanxi Province, China, during the 2018 and 2019 spring–summer seasons.

## 2. Materials and Methods

### 2.1. Experimental Sites

The study was conducted in two consecutive seasons, 2018 and 2019, and within two different *Actinidia chinensis* var. *deliciosa* ‘Hayward’ kiwifruit orchards, one organic (unsprayed) and one conventional (sprayed), in Mei County, Shaanxi Province, China. The organic orchard, with an area of 12,800 m^2^ (80 × 160 m^2^), was located at the Northwest Agriculture and Forestry University kiwifruit experimental field station (34°07′27″ N; 107°59′31″ E). The conventional orchard, with an area of 1700 m^2^ (10 × 170 m^2^), was located 2000 m away from the organic orchard. In the conventional orchard, six chemical sprays were applied during the 2018 kiwifruit growing season: two applications of cyhalothrin (May and June), and one application each of fenvalerate (June), clofentezine (June), λ-cyhalothrin (September), and chlorpyrifos (September). In 2019, two cyhalothrin sprays (April and June) and one of bifenthrin (May) were applied.

### 2.2. Egg Parasitoid Surveys in Kiwifruit Orchards

Egg parasitoid surveys were conducted at each site by exposing laboratory-reared ‘sentinel’ BMSB egg masses at different times from May to October. The BMSB colonies were maintained in nylon mesh cages (60 × 60 × 60 cm), and reared continuously on a diet of fresh ears of corn (*Zea mays* L.) and green bean pods (*Phaseolus vulgaris* L.) in a controlled temperature and humidity room set at 25 ± 1 °C, 65 ± 5% RH and a 16:8 h [light:dark] cycle. Each cage was provided with water in wet cotton wool and a folded piece of wax paper as a substrate for oviposition. Sentinel egg masses to be used in the field trials were collected daily from BMSB laboratory colonies and mounted onto clear glue (Haijiaowang, Shanxi Haijiao Science, Industry and Trade Co., Ltd., China) on a 60 × 20 cm cardboard strip. The number of eggs in each egg mass was recorded on the cardboard strip.

In 2018, sentinel egg masses were deployed and exposed to wild parasitoids once a month, from 16 June to 12 October, at each site. During each deployment, no fewer than ten fresh (i.e., one to three day-old) sentinel BMSB egg masses (average 26 eggs/mass) were attached to the underside of leaves of randomly selected kiwifruit vines within the orchard. Exposed sentinel egg masses were marked with colour flagging tape attached to the branch of the vine carrying the egg mass. Sentinel egg masses were retrieved after three to five days (depending on weather/temperature). Retrieved egg masses were individually maintained in small petri dishes (60 mm × 15 mm) and stored at 25 ± 1 °C, 65 ± 5% RH and a 16:8 h [light:dark] cycle until all parasitoids and/or BMSB nymphs had emerged.

The same procedure was conducted during 2019 except for the frequency of the deployment of egg masses. Because of the availability of a high number of sentinel egg masses during the second season, sentinel egg masses were deployed twice a month at each site. Based on the 2018 parasitoid survey results, the survey starting dates in 2019 were shifted to May, to include the assessment of parasitoid presence in early spring.

All emerged parasitoids recovered from each retrieved BMSB egg mass were counted and transferred into glass vials with 80% ethanol for morphological identification. Unhatched eggs were also counted and dissected under a stereomicroscope. After dissections, unhatched eggs were labelled as either unemerged parasitoid (i.e., pharate adults observed), unemerged BMSB nymph (i.e., undeveloped nymph observed), or unviable eggs (i.e., undetermined content). Information on emerged and unemerged parasitoids was used to determine observed parasitism (i.e., number of sentinel BMSB egg masses recovered from which at least one emerged or unemerged parasitoid was recorded) and successful parasitism (i.e., number of emerged parasitoids recorded from parasitised BMSB eggs).

### 2.3. Parasitoid Identification

All parasitoids were identified using a stereomicroscope following Talamas et al. [43] and Peng et al. [44]. The Acroclisoides sp. found during the 2019 survey was identified, to the genus level only, by Hui Xiao (Institute of Zoology, Chinese Academy of Sciences, Beijing, China).

### 2.4. Data Analysis

A generalised linear model (GLM) with binomial distribution was used to investigate differences between orchard management systems and observed parasitism in the 2018 and 2019 surveys. The fixed effects were management systems, experimental months, and their interactions. Binary variates (yes or no) of observed parasitism were fitted to the GLM model in each season separately. Owing to the lack of spatial replication, we note that although the management system was a fixed effect, its statistical significance cannot be generalized but is seen as a preliminary indication only on the potential effect of management systems on observed parasitism. Months where no parasitism was recorded were excluded from the analysis.

The number of *T. japonicus* out of the binomial total of the emerged parasitoids (=successful parasitism) was fitted to the GLM model to investigate the potential impacts of the site (within management system), month, and their interactions on *T. japonicus* abundance among other parasitoids. A similar analysis was performed for observed abundance of *T. cultratus* (Mayr). The same circumstances relating to the confounding effect of the management system and site location hold in this case. The relative abundances of *T. cultratus* and *T. japonicus* were mutually dependent, as increasing one decreased the other. Therefore, the relative abundance of one can be deduced from the other, especially if the other species of parasitoids the emerged were deficient. The design and variation in the data restricted the performance of formal multinomial analysis to measure proportional probabilities.

All computations and graphics were performed using R in R Studio platform, version 1.1.422, using lme4, predictmeans, and ggplot2 packages (https://cran.r-project.org; accessed on 10 June 2020).

## 3. Results

### 3.1. Seasonal Parasitism of H. halys Sentinel Egg Masses

Totals of 2932 sentinel BMSB eggs from 112 egg masses (2018), and 6303 eggs from 233 masses (2019) were deployed and exposed to parasitism in kiwifruit. Sentinel eggs retrieved from the field (2902 in 2018 and 6254 eggs in 2019) confirmed parasitism occurred from June to August, and from May to August, across the two survey seasons respectively. During 2018, mean observed parasitism across both orchards from June to August was 48%, and parasitism reached 54% in June. However, overall observed parasitism did not differ significantly (*p*-value > 0.05) between the different months. The 2019 survey recorded observed parasitism of 29% across both orchards from May to August, reaching 34% in August. Similarly to 2018 surveys, overall mean observed parasitism in 2019 did not differ significantly (*p*-value > 0.05) between the different months across the two orchards.

**Figure 1:**
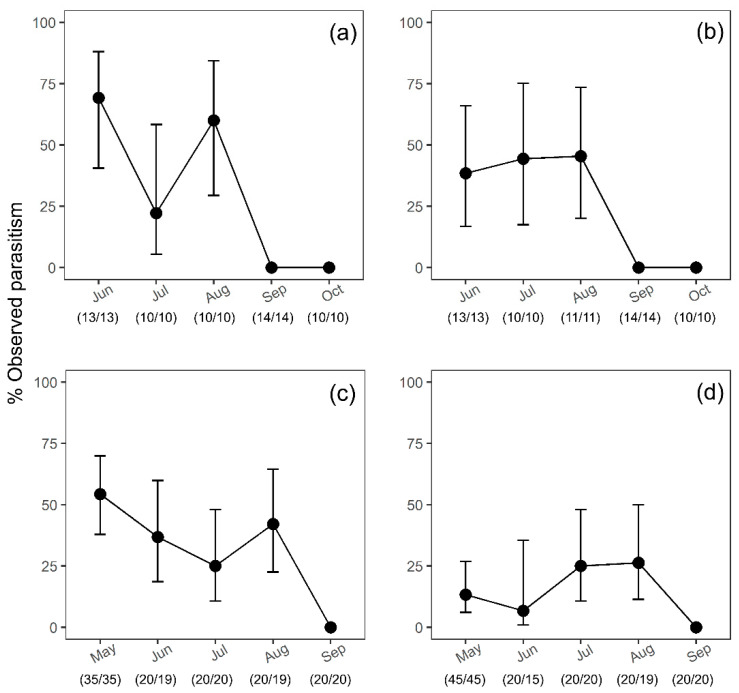
Estimated parasitism (with 95% confidence intervals) from sentinel *Halyomorpha halys* egg masses exposed to natural parasitism in Mei County, Shaanxi Province, China during egg parasitoid surveys in kiwifruit in: (**a**) 2018 organic orchard, (**b**) 2018 conventional orchard, (**c**) 2019 organic orchard, and (**d**) 2019 conventional orchard. Black circles are the back-transformed predicted means by the generalised linear model (GLM) model; the vertical lines show the back-transformed confidence intervals. Numbers in brackets indicate the number of egg masses exposed/retrieved. Overlapping confidence intervals for parasitism suggest no evidence of a statistically significant difference.

In 2018, observed parasitism in the organic orchard (53%) appeared slightly higher than that observed in the conventional orchard (42%). No statistical evidence was found to suggest differences in observed parasitism between organic and conventional kiwifruit orchards, among the months or their interactions (all *p*-values > 0.05) (Figure 1a,b). However, in the organic orchard, observed parasitism in June and August was nearly three times higher than that observed in July (Figure 1a). There was, however, very high variability in samples contributing to these mean values.

Observed parasitism in 2019 organic and conventional kiwifruit orchards between May and August averaged 42% and 17%, respectively. Similarly to surveys in 2018, no parasitism occurred in September in either orchard. Statistical analysis suggests a significant difference in observed parasitism between organic and conventional kiwifruit orchards in 2019 surveys (*p*-value < 0.001), but no significant differences in parasitism were noted between different months (*p*-value > 0.05) (Figure 1c,d). In contrast, in the conventional orchard, the mean observed parasitism in July (25%) and August (26%) nearly doubled those observed in May and June (Figure 1d), albeit again with the same high variability recorded in 2018.

**Figure 2:**
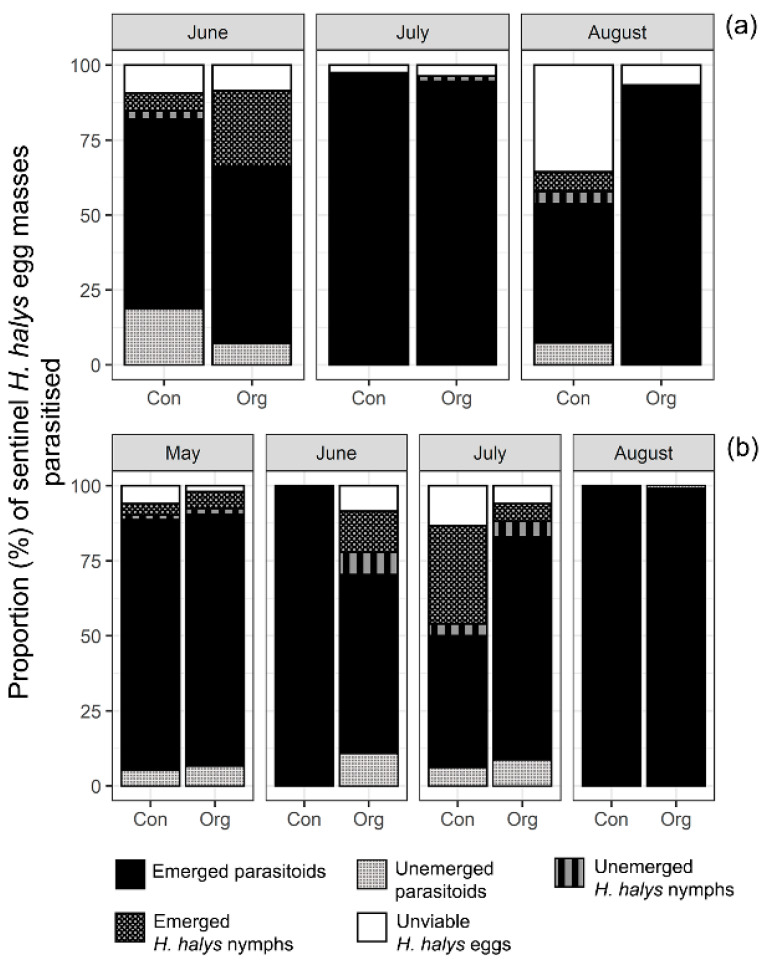
Relative proportion of parasitoids and *H. halys* nymphs development per parasitised egg masses (raw data) recovered in organic (Org) and conventional (Con) kiwifruit orchards in Mei County, Shaanxi Province, China during egg parasitoid surveys in: (**a**) 2018 and (**b**) 2019. Arithmetic means are shown without standard errors (SEs) to allow visualization, as the underlying distribution are not Normal.

High rates of emerged adult parasitoids (=successful parasitism) were recorded from parasitised eggs recovered from both 2018 and 2019 surveys in both organic and conventional kiwifruit orchards. In 2018, in the organic orchard, the average percentage of emerged parasitoids was highest in July and August, with 95% and 93% emergence recorded, respectively (Figure 2a). In the conventional orchard, the highest average percentage of emerged parasitoids was recorded in July (98%) (Figure 2a). In 2019, the highest parasitoid emergence in both organic and conventional orchards was found in May and August (Figure 2b). In May, mean emerged parasitoids were 89.2% and 100%, in organic and conventional orchards, respectively, whereas in August, this was 99% in both orchards (Figure 2b). In June 100% of observed parasitoids developed in the conventional orchard (Figure 2b).

### 3.2. Parasitoid Species Recovered and Abundance

**Figure 3:**
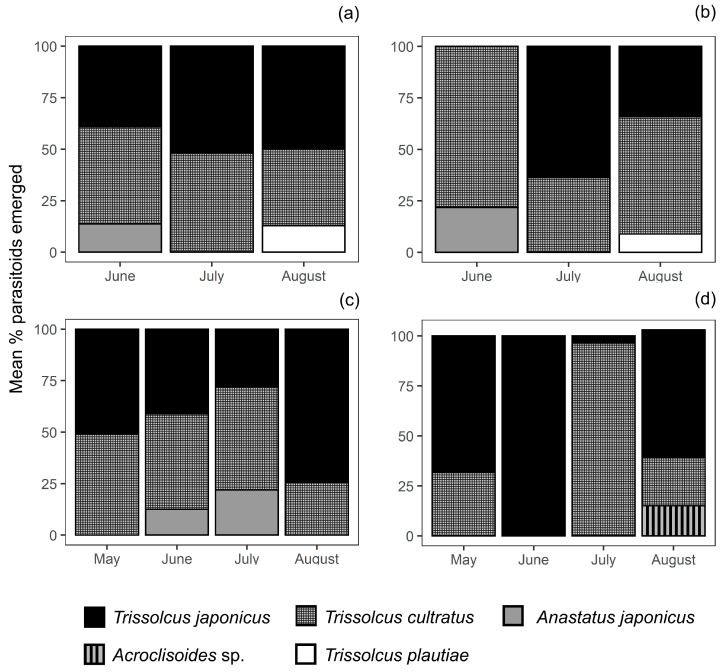
Relative abundance of parasitoid species (raw data) recovered from successfully parasitised sentinel *Halyomorpha halys* egg masses deployed monthly in Mei County, Shaanxi Province, China during egg parasitoid surveys in kiwifruit in: (**a**) 2018 organic orchard, (**b**) 2018 conventional orchard, (**c**) 2019 organic orchard, and (**d**) 2019 conventional orchard. Arithmetic means are shown without standard errors (SEs) to allow visualization, as the underlying distribution are not Normal.

During the 2018 and 2019 surveys, 1816 parasitoids (588 and 1228, respectively) emerged from parasitised sentinel BMSB eggs masses retrieved. On average overall, 77% emergence was confirmed from successfully parasitized egg masses across the two orchards in the two-year surveys. In 2018, four parasitoid species were recovered during the survey season: *T. japonicus*, *T. cultratus*, *T. plautiae* (Watanabe), and *Anastatus japonicus* Ashmead. *Trissolcus japonicus* and *T. cultratus* were the two predominant species in both organic (Figure 3a) and conventional orchards (Figure 3b). *Trissolcus japonicus* and *T. cultratus* accounted for an overall average of 41% and 48% of relative abundance across the two orchards, respectively, while *A. japonicus* for 7% and *T. plautiae* for 4%.

Four parasitoid species were also recovered during the 2019 survey season. However, during 2019, *T. plautiae* was not recorded and an additional species (*Acroclisoides* sp.) was recovered instead. Similarly to 2018 surveys, *T. japonicus* and *T. cultratus* were the two predominant species recovered in organic (Figure 3c) and conventional (Figure 3d) orchards. Across the two kiwifruit orchards, *T. japonicus* and *T. cultratus* accounted for an average of 55% and 41% emerged parasitoids, respectively, while *A. japonicus* for 3% and *Acroclisoides* sp. for 2%.

### 3.3. Relative Abundance of T. japonicus and T. cultratus in Kiwifruit Orchards

In 2018, *T. japonicus* accounted for 41% (243) of emerged parasitoid species across the surveyed orchards, whereas *T. cultratus* accounted for 48% (285). Monthly *T. japonicus* abundance differed significantly (*p*-value < 0.001), with a peak in abundance in July (60%). *Trissolcus* *japonicus* was slightly more abundant than *T. cultratus* in July and August only. Similarly, abundance of *T. cultratus* also differed significantly (*p*-value < 0.001) between months, but its peak abundance (59%) was observed in June (See Supplementary Figure S1 for estimated abundance model). Surveys conducted in 2019 showed that *T. japonicus* and *T. cultratus* accounted for 55% (670) and 41% (502) of developed parasitoid species recovered across the orchards, respectively. Similar to 2018, parasitism data showed that *T. japonicus* differed significantly (*p*-value < 0.001) between different months, with peaks in abundance during May–June (56–52%) and then in August (70%). Monthly abundance of *T. cultratus* also differed significantly (*p*-value < 0.001), with its peak abundance (67%) in July (See Supplementary Figure S1 for estimated abundance model).

**Figure 4:**
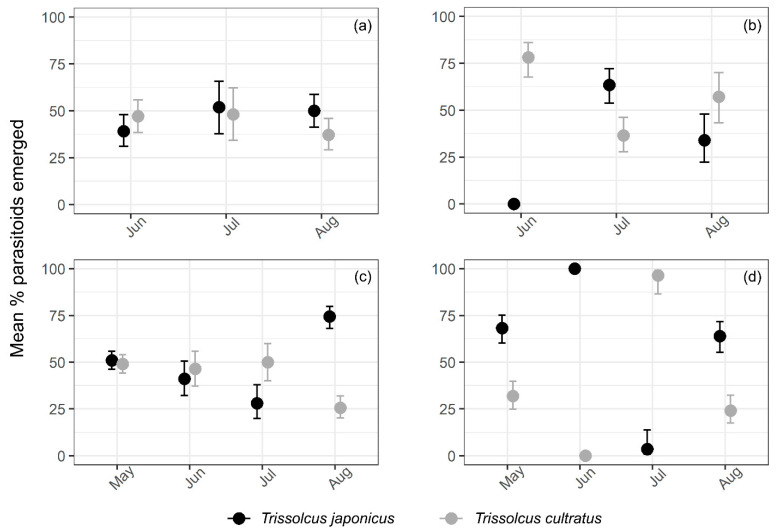
Estimated mean abundance of *Trissolcus japonicus* and *T. cultratus* (with 95% confidence intervals) recovered from successfully parasitised sentinel *Halyomorpha halys* egg masses in Mei County, Shaanxi Province, China during egg parasitoid surveys in kiwifruit in: (**a**) 2018 organic orchard, (**b**) 2018 conventional orchard, (**c**) 2019 organic orchard, and (**d**) 2019 conventional orchard. The circles are the back-transformed predicted means by the generalised linear model (GLM) model and the vertical lines show back-transformed confidence intervals. Non-overlapping confidence intervals within each species suggest evidence of statistically significant difference.

In the organic orchard, *T. japonicus* was overall slightly more abundant (46%) than *T. cultratus* (43%) during the 2018 survey season. However, in the conventional orchard, the opposite was observed, *T. cultratus* being overall more abundant (55%) than *T. japonicus* (36%). Monthly recorded data from the 2018 survey revealed that the abundance of *T. japonicus* in the organic orchard was very similar from June to August (39–52% range) (Figure 4a), while it was slightly more abundant (63% average) in July in the conventional orchard (Figure 4b). Overall, *T. japonicus* was significantly more abundant (*p*-value < 0.001) in the organic orchard than the conventional orchard, and its abundance differed significantly (*p*-value < 0.001) between months in the conventional (abundance was highest in July—64%) but not the organic orchard (Figure 4a,b). *Trissolcus cultratus* abundance did not differ significantly (*p*-values > 0.05) between the organic and conventional orchards, and varied slightly (37–48% range) across the season in the organic orchard (Figure 4a). However, in the conventional orchard, *T. cultratus* abundance varied significantly (*p*-value < 0.001) across the survey season, being significantly more abundant (78%) in June (Figure 4b).

During the 2019 survey season, *T. japonicus* was overall slightly more abundant (53%) than *T. cultratus* (43%) in the organic orchard. Similar observations were recorded for the conventional orchard, with *T. japonicus* accounting for 58.4% of parasitoids overall abundance versus 36% for *T. cultratus*. *Trissolcus japonicus* was significantly more abundant (*p*-value < 0.001) in August (74% average), followed by May (51% average) and June (41% average) in the organic orchard (Figure 4c). However, in the conventional orchard, overall abundance of *T. japonicus* was significantly higher (*p*-value < 0.001) in June (100%), followed by May (68%) and August (62%) (Figure 4d). In contrast to the 2018 season, *T. japonicus* abundance did not differ significantly (*p*-value > 0.05) between the organic orchard and the conventional orchard in 2019. The abundance of *T. cultratus* in the organic orchard varied slightly within the first three months of the 2019 season (46–50% range), and was significantly lower (*p*-value < 0.001) in August (27%) (Figure 4c). *Trissolcus cultratus* overall abundance differed significantly (*p*-value < 0.001) between months in the conventional orchard, being highest in July when accounted for 96% of observed parasitism (Figure 4d). However its abundance did not exceed 30% in the other surveyed months and it was completely absent from samples collected in June (Figure 4d). Similarly to *T. japonicus*, the abundance of *T. cultratus* did not differ significantly (*p*-value > 0.05) between the organic orchard and the conventional orchard in 2019.

## 4. Discussion

Biological control is considered a key component of integrated pest management programmes. It is regarded as a highly cost-effective long-term pest control method, and is often considered the only way to establish and maintain a self-sustaining reduction of insect pests [45,46,47]. Many years of research have been conducted on potential biocontrol options against BMSB, both in its invaded and native ranges [1,15,18,48]. Several resident parasitoid natural enemies have been identified attacking BMSB in its adventive ranges, but these have shown little or no promise for use in a biocontrol programme [15,49,50]. However, surveys in its native range in China, have identified *T. japonicus* as the most promising biocontrol agents for BMSB [18,20].

Our two-season study provides the first multi-season survey results of BMSB egg parasitism in kiwifruit orchards. Additionally, we report new information on the community composition of parasitoids associated with BMSB in kiwifruit. More than 9000 sentinel BMSB eggs (i.e., >300 egg masses) were exposed to natural parasitism across two survey seasons in two different kiwifruit orchards. Results for parasitoid species in our study are consistent with previous reports, where observed parasitism on sentinel BMSB egg masses deployed across native forest sites and in different fruit trees in mixed orchards averaged 34.7% [18]. However, overall rates of observed parasitism in our study were much lower than those reported by Zhang et al. [18] from naturally laid BMSB egg masses collected from agricultural and native forest sites in Beijing, and also by Benvenuto et al. [51] in organic kiwifruit orchards in north-eastern Italy, which averaged 78.8% and 84%, respectively. Therefore, the differences in observed parasitism between our study and those reported in naturally laid BMSB egg masses in China and Italy [18,51] suggest that sentinel egg mass parasitism is likely to be underestimating actual parasitism in the field. While data collected from naturally laid egg masses is probably more accurate, the availability of such data is often limited.

The abundance of *T. japonicus* in our study is consistent with the reports of previous studies in its native range in northern China by Qiu, et al. [52] and Yang et al. [20], where parasitism by *T. japonicus* averaged 50%. However, our results differ from survey results from Beijing, where *T. japonicus* was found to be the predominant parasitoid species recovered across agricultural and native forest sites from both sentinel BMSB egg masses (average 90% parasitism) and naturally laid eggs (average 77% parasitism) [18]. Parasitism by a given species may vary depending on the environmental and ecological contexts. The reported abundance of *T. japonicus* and *T. cultratus* in our study corresponds to survey results in kiwifruit orchards. In contrast, data from Zhang et al. [18] comes from surveys in several different host plants and environments, such as peach, mulberry, jujube, and native trees. In similar parasitoid surveys conducted in apple and peach orchards in New Jersey, USA, to assess the presence of adventive *T. japonicus*, parasitism occurred at low rates in peach orchards, where the majority of developing parasitoids (i.e., 97.4%) from successfully parasitised sentinel egg masses were *T. japonicus*. No parasitism was recorded in apple orchards [42]. However, this study is the first report of *T. japonicus* in North American agriculture, and the low parasitism rates observed are likely due to the early stage of the parasitoid establishment. Peach is known as one of the preferred host plant of BMSB [53], where it frequently occurs in high numbers. This may increase the likelihood of encounters with particular egg parasitoids (e.g., *T. japonicus*), where competition could reduce the abundance of other less effective parasitoids, such as *T. cultratus*. *Trissolcus* species (including *T. japonicus*) have been reported to be more predominant in wooded habitats [16,29,54,55], and there are data that show that *T. japonicus* emerges most frequently from BMSB egg masses found at mid-canopy [41].

A recent study, that looked at the presence of BMSB egg parasitoids by collecting and rearing naturally-laid BMSB egg masses in a single kiwifruit orchard in August 2018 and 2019 in north-eastern Italy, found very high parasitism rates on BMSB eggs (i.e., 87–88% average each year) from parasitized BMSB egg masses collected [51]. The parasitoid *Trissolcus mitsukurii* (Ashmead), which was first found in Italy along with *T. japonicus* in 2018 [27], was the predominant parasitoid (over 97% parasitism) in both years [51]. The hyperparasitoid *Acroclisoides sinicus* (Huang and Liao) was also observed, but in very small numbers (i.e., 1.9% average parasitism) and only in 2019 [51]. In our study, we did not find *T. mitsukurii* in the surveyed kiwifruit orchards in China. However, the initial findings of high parasitism rates by *T. mitsukurii* in kiwifruit reported by Benvenuto et al. [51] are comparable to those found for *T. japonicus* in China [18], and provide us with promising results of an additional candidate parasitoid that could be used in a classical biocontrol against BMSB in kiwifruit orchards.

In our survey, the abundance of *T. japonicus* varied throughout the season in both kiwifruit orchards in both seasons, with a 2019 bimodal distribution of parasitism peaking in May–June and another one in August. This is consistent with *T. japonicus* reports in northern China, where two peaks have been observed in May–June and early August, mirroring BMSB peaks [52]. However, our 2018 survey showed a unimodal distribution of parasitism that peaked in July. A similar parasitism pattern of exposed sentinel BMSB egg masses was observed during two consecutive years in northern China near Beijing, where parasitism was observed to increase from early season, and peak in August [18].

Several studies show the potential impacts of insecticides on parasitism and survival of egg parasitoids [56,57,58,59]. Recent studies reported that the survivorship of female *T. japonicus* could be negatively affected when exposed to BMSB egg masses sprayed with bifenthrin, as well as clothianidin [59,60]. Also, significant reductions in adult *T. japonicus* emergence were reported by Ludwick et al. [59] from egg masses recovered from some areas sprayed with methomyl, thiamethoxam + λ-cyhalothrin, and bifenthrin. Parasitized egg masses exposed to insecticides could also result in a reduction of parasitoids emergence [57,58,61]. During our parasitoid surveys, parasitism was found to differ significantly between the organic and conventional orchards only in 2019, where two applications of cyhalothrin (April and June) and one of bifenthrin (May) were made. Interestingly, during 2018 surveys, chemical pesticides were sprayed more often in the conventional orchard during 2019 surveys, but observed parasitism did not differ significantly from that in the organic orchard. However, bifenthrin was not used during 2018. *Trissolcus japonicus* was significantly more abundant in the organic than the conventional orchard in 2018. In contrast, in 2019 surveys, its abundance did not differ significantly between organic and conventional orchards. In *T. cultratus*, no differences were found in its abundance between the two orchards during the 2018 and 2019 surveys. The differences in overall parasitism recorded in 2019 and *T. japonicus* abundance in 2018 between organic and conventional orchards could be associated with adverse effects caused by insecticides sprayed during the season. However, differences between management systems detected in our study could also result from seasonal or site effects rather than an effect of management systems, as we surveyed only one orchard of each management type in our two-season study. Thus, interpretation of these results needs to be undertaken cautiously and the results viewed only as an indication of the potential effects of management systems on parasitism and species abundance. More surveys in other organic and conventional kiwifruit orchards are needed to provide more supporting evidence of any effects of management systems on parasitism and species abundance. Nevertheless, although *T. japonicus* can survive in sprayed crops to some extent [28,42], differences in its performance and abundance are likely to vary between sprayed and unsprayed crops.

## 5. Conclusions

In summary, our two-year survey study conducted in two green-fleshed ‘Hayward’ kiwifruit orchards (i.e., one organic and one conventional) in Mei County, Shaanxi Province, China, showed that parasitism occurred between May and August. Parasitism rates fluctuated across orchards between the two survey years (i.e., 48% in 2018, and 29% in 2019), and in both years *T. japonicus* and *T. cultratus* were confirmed as similarly abundant. Although no definite conclusions can be made on the effect of management system on the abundance and diversity of BMSB egg parasitoids (i.e., due to lack of spatial replication), our results provide preliminary data on the potential effect of management systems on observed parasitism.

Our study provides the first multi-season survey of egg parasitoids of *H. halys* in ‘Hayward’ kiwifruit orchards. Results of this study not only provide valuable information on the season-long abundance and diversity of egg parasitoids attacking *H. halys* in kiwifruit, but also provides some insight into the abundance and relative effectiveness of *T. japonicus* in kiwifruit orchards under different management systems. This additional knowledge is expected to contribute to the planning of future releases of *T. japonicus* as part of a classical biological control programme for BMSB in kiwifruit.

## Data Availability

Not applicable.

## Supplementary Materials

The following are available online at https://www.mdpi.com/article/10.3390/insects12050428/s1, Figure S1: Estimated abundance of *T. japonicus* and *T. cultratus* (with 95% confidence intervals) recovered from parasitised sentinel *H. halys* egg masses in Mei County, Shaanxi Province, China during egg parasitoid surveys in (a) 2018 and (b) 2019. The circles are the back-transformed predicted means by the GLM model and the vertical lines show the back-transformed confidence intervals. Non-overlapping confidence intervals within each species suggest evidence of statistically significant difference.
