# Supplementary material for: Seasonal Abundance and Diversity of Egg Parasitoids of Halyomorpha halys in Kiwifruit Orchards in China
Source: Insects. 2021 May 10;12(5):428. doi: 10.3390/insects12050428 (PMC8151322; doi:10.3390/insects12050428)
Supplement: Supplementary file 1 [file insects-12-00428-s001.zip › insects-1183014-supplementary.pdf]

insects

Supplementary Material: Seasonal Abundance and Diversity of Egg Parasitoids of *Halyomorpha halys* in Kiwifruit Orchards in China

Gonzalo A. Avila, Juhong Chen, Wenjing Li, Maryam Alavi [1], Qianqian Mi, Manoharie Sandanayaka, Feng Zhang and Jinping Zhang

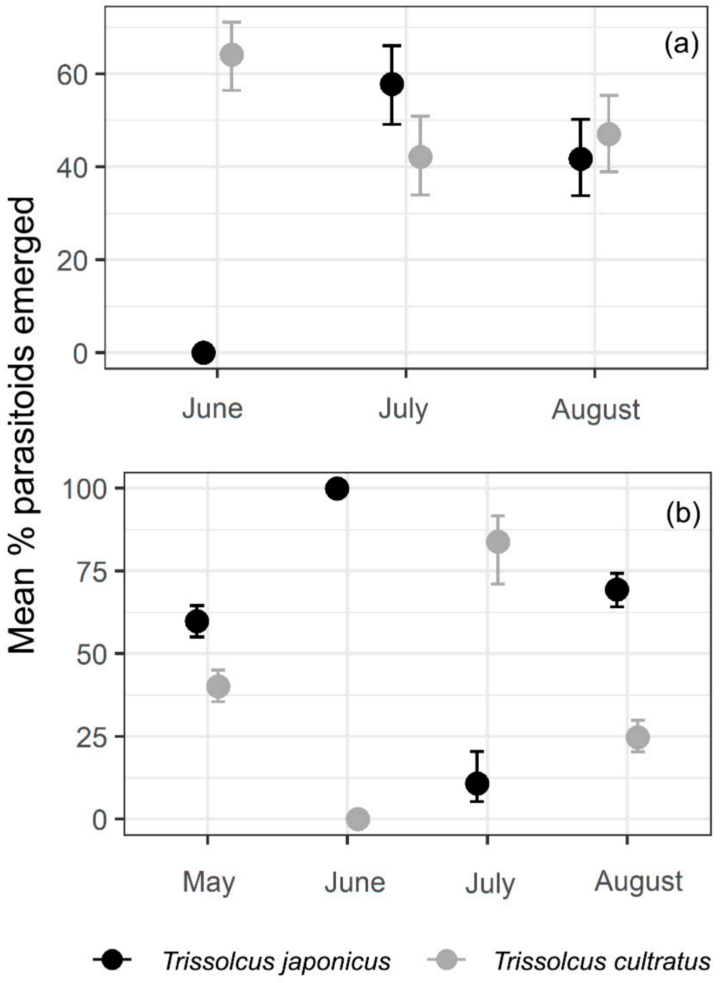

Figure S1. Estimated abundance of *T. japonicus* and *T. cultratus* (with 95% confidence intervals) recovered from parasitised sentinel *H. halys* egg masses in Mei County, Shaanxi Province, China during egg parasitoid surveys in (**a**) 2018 and (**b**) 2019. The circles are the back-transformed predicted means by the GLM model and the vertical lines show the back-transformed confidence intervals. Non-overlapping confidence intervals within each species suggest evidence of statistically significant difference.
